# Review of Some of the Surgical Cases Which Have Lately Occurred in the Royal Infirmary of Edinburgh. A Clinical Lecture Delivered to the Students of Surgery in That Institution, on Thursday, 26th February, 1829

**Published:** 1829-07

**Authors:** George Ballingall

**Affiliations:** Fellow of the Royal College of Surgeons, Surgeon Extraordinary to the King, Regius Professor of Military Surgery in the University of Edinburgh, and One of the Surgeons to the Royal Infirmary.


### DR. BALLINGALL'S CLINICAL LECTURE.

*Review of some of the Surgical Cases which have lately occurred in the* Royal Infirmary *of* Edinburgh. *A Clinical Lecture delivered to the Students of Surgery in that Institution, on Thursday, 26th February,* 1829, *by* George Ballingall, M.D. F.R.S.E.; Fellow of the Royal College of Surgeons, Surgeon Extraordinary to the King, Regius Professor of Military Surgery in the University of Edinburgh, and one of the Surgeons to the Royal Infirmary.

Gentlemen: In commencing a retrospect of the fifth course of Clinical Lectures, which, as the senior attending surgeon of the house, it has become my duty to conclude, I would, in the first place, direct your attention to some of the accidents and acute cases which you have had an opportunity of witnessing during the last four months.

Of the acute cases, the fractures of the limbs have, upon the present, as upon former occasions, constituted a large majority; of simple fractures I find that there have been treated, on my side of the house, twenty-six cases, all of which have either terminated successfully or are in the progress of cure, with the exception of that of William Fernie, æt. sixty-two, who was admitted on the 3d of January, with a fracture of the neck of the femur. This man was brought from Kinglassie in Fifeshire, where he had met with the accident about ten days before. His limb was immediately placed in Boyer's splint, and was extended to the same length as its fellow on the opposite side; but, although the old man complained of no inconvenience from the apparatus, it was soon perceived that ulceration had taken place on the sacrum to a considerable extent. This induced me to remove the splint, and to place the patient on his side. Here again a gangrenous spot appeared over the trochanter of the right thigh, and the patient was subsequently turned to the opposite side; my object now being to obviate the effects of pressure and the extension of the gangrene, rather than to look for any successful treatment of the fracture. The ulceration, however, continued to extend, and the patient's strength to sink, notwithstanding the liberal exhibition of animal food, porter, and wine, until the 4th of February, when he died, six weeks after the receipt of the injury; and I regret to say that the body was immediately removed to Fife, without affording us an opportunity of examining the state of the broken bone.

In the cursory observations which I have had occasion to lay before you on the subject of fractures, I have always inculcated the necessity of giving your attention to the general principles on which fractures are treated, observing that the variety of these accidents is so endless as to render it a matter of impossibility to be previously acquainted with the specialities of each individual case. My views of the treatment of fractures of the neck of the femur I explained to you from one of my printed Lectures; and I would now only observe, that the result of the foregoing, as well as of some other cases of the same kind which I have witnessed, has made me lately disposed to question whether we would not do well, in many cases of fracture of the neck of the thighbone, to be satisfied with the simple treatment recommended by Sir A. Cooper: to place the limb in a relaxed position, with a folded pillow under the knee-joint, and to take our chance of such a cure as nature may be pleased to afford us, rather than, by confining the patient in a coercive apparatus, to run the risk of inducing ulceration or gangrene; the progress of which, in advanced life, or in broken constitutions, it is so little within our power to control.

Of the cases of compound fracture occurring during the present course, that of Margaret Thomson, æt. seventy-four, who was admitted on the 3d of November, having both bones of the fore-arm broken, terminated fatally with symptoms of effusion into the thorax, four days after her reception into the hospital.

A case of compound fracture of the leg presented itself in the case of John Hamilton, æt. fourteen, who was under treatment in the early part of the season, in whom the cure was considerably advanced previous to the commencement of the course, and who was eventually dismissed cured on the 21st of December, after a confinement of upwards of twelve weeks.

The only other case of compound fracture which has been under my care during the present session was that of Alexander Kerr, æt. twenty, whose case was of a very severe and dangerous character, and of which the following particulars are recorded:

"Admitted, October 6th.—The whole left leg is very much swelled, tense and painful. About an inch and a half above the ankle, there is a small wound upon the anterior surface of the tibia, and the skin above the outer malleolus is very much contused, and fluctuates from the effusion of blood beneath it; when the foot is forcibly moved, an indistinct crepitus is felt. About eight o'clock this morning he was overturned in a cart, the edge of which fell against the lower part of his leg, whilst the weight of the horse was resting against the shaft. Limb was placed on M'Intyre's splint, and the spirit lotion applied."

Took an anodyne draught at bedtime, and a dose of oil on the following morning. The spirit lotion was continued to the leg, and an antimonial solution administered internally from time to time. On the 8th, gangrene began to appear, and the following report was entered:

"Slept ill; pulse 108, very full and strong; bowels once relieved; tongue slightly furred; skin upon the outer side of the leg is evidently gangrenous, and that upon the inner side considerably discoloured. An incision was made through the former, some serum was discharged, but the wound bled very little. He was bled to twenty ounces.

"Eleven o'clock.—Pulse 132; gangrene does not appear to have spread on the outside, but the skin upon the inside is more extensively discoloured.—V.S. ad ℥x.

"9th.—Slept a little; pulse 112, not so strong; skin hot; less thirst; bowels once relieved yesterday; gangrene has not spread at all upon the outside of the limb, and there is some healthy purulent discharge from the incision made yesterday; on the inside, the gangrene has certainly spread a little. Last blood drawn neither buffed nor cupped."

The fermenting poultice was applied to the leg, and the antimonial solution continued internally. His feverish symptoms now began to subside, and on the 11th it was reported that "his pulse was 100; skin cool, tongue less furred, appetite returning; gangrene had ceased to spread, but the sloughs had not begun to separate."

It now became obvious that we should not be compelled to amputate by the extension of the gangrene, but, when sloughs came to be detached, and both the tibia and fibula were laid bare to a considerable extent, with a copious purulent discharge, and rather profuse sweats, I was greatly inclined to amputate the leg; and, had not some of my colleagues entertained a more favorable opinion of the case than I did, it is probable the operation would have been proposed to him.

There was great encouragement, however, to persevere in our attempts at a cure, from the prosperous state of the man's general health, as well as from his youth and apparently vigorous habit. The extensive sore which nearly surrounded the lower part of the limb became covered with florid and healthy granulations; the discharge rapidly diminished; and on the 21st December he was discharged cured, a trifling exfoliation having previously taken place from the fore part of the tibia.

Of compound fractures I have hitherto said but little in these lectures, nor do I now propose to enlarge; for, although in the two cases just mentioned you have witnessed a successful termination under the treatment adopted, yet they are, upon the whole, a class of accidents, the results of which have been to me the least satisfactory of all that I have had occasion to treat in this house, and are not coincident with my experience of them in other situations.

Had these results been the consequence of one uniform mode of treatment, I should naturally have concluded that that treatment was erroneous. But, when I look back to the mode of dressing the wounds, sometimes by a piece of lint soaked in blood, and allowed to form an incrustation over them; sometimes by covering them with a paste of gum or a pledgit of simple dressing, and sometimes by bringing their lips into accurate apposition with adhesive straps: when I look again to the different positions in which the limb has been placed, either closely enveloped in splints, lying less constrained in a fracture box, or simply resting on a pillow, sometimes in the bent, sometimes in the extended position; and when I look also to the various means taken to subdue the violent inflammation and high symptomatic fever accompanying these injuries; by general or by local bleeding, according to circumstances; very frequently by the use of cold evaporating lotions to the seat of the injury, and sometimes by the use of anodyne fomentations or cataplasms: I cannot admit that the unfavorable results which I have so often had occasion to deplore have been attributable to any bigoted prejudices or exclusive partialities in the mode of treatment.

In my remarks upon this subject, I took occasion to observe that we are greatly in want of a work on these accidents, from some surgeon of varied and extensive experience. I say varied experience, because it appears to me that we are often led, by the irresistible force of habit, to give our attention too exclusively to one mode of treatment. I remember to have heard an hospital surgeon assert that he never expected to lose another case of compound fracture, by following up the practice, which seemed to him to be a new one, of closely enveloping such fractures in splints and bandages, without undoing them for weeks together.* But it is not from those who take such a limited or exclusive view of this matter that we are to expect such a work as I could wish to see, but from those who are capable of discriminating between those cases (perhaps numerous ones) in which the above practice is advantageous, and those in which it is not only injurious, but absolutely insufferable.

Before quitting this subject, I would beg leave to mention that there are two points in the treatment of compound fractures upon which my own observation has led me to form a very decided opinion, and to offer you a remark which may possibly prove useful hereafter. I have, I think, too frequently seen a reluctance to use the saw in removing the protruding extremities of the bone, when these were either difficult to reduce or of a sharp and spicular form; and I have, I think, sometimes seen the closure of the external wound attempted by means too forcible and too long continued.

Among the more severe accidents requiring amputation

* The Orientals adopt a somewhat similar mode of practice. Vide London Med. and Phys. Journal for February 1829, p. 175. Baron Larrey, we believe, treats simple and compound fractures in this manner.

EDITORS.

which have occurred during the present course, that of Robert M'Gregor, æt. fifteen, deserves particular attention. This lad was admitted late on the evening of the 28th of January, and the following notice of his case entered in the journal:

"Had his right arm drawn into the machinery of a paper mill at five P.M. The arm was torn off from the body above the middle of the humerus. The extremity of the bone projected out from the muscles. The laceration extends on the inner side of the arm into the axilla, destroys the inferior margin of the pectoralis major and latissimus dorsi muscles. The skin, for a short way on the back of the scapula is destroyed, and also a considerable part of the clavicular portion of the deltoid.

"The artery is seen pulsating about three inches from its end, which is closed by coagulated blood. No hemorrhage had taken place; his extremities were cold, and pulse feeble."

The operation at the shoulder-joint was performed by Dr. CAMPBELL, who succeeded me in the active duties of the house. A single flap was made from the deltoid, which completely covered the wound; four vessels were secured, and an opiate administered. The patient had a good night after the operation, and no untoward circumstance occurred until the night between the 1st and 2d of February, when a secondary hemorrhage took place, which is thus noticed in the journal:

"About ten P.M. there came on a considerable hemorrhage from a small vessel, which was tied; and, about half past two this morning, the ligature came away from the axillary artery, and hemorrhage ensued. The vessel was immediately secured; a considerable quantity of blood was lost. Pulse about 130, stronger than yesterday evening. An anodyne was given.—Cont. H. Anodyn. cum T. Opii, gtt. xxxv."

For several days after this the patient appeared to be in considerable hazard: some sloughing took place from the edges of the wounds, with a copious discharge of ill-conditioned matter; his pulse varying from 125 to 150, with occasional rigors and profuse sweatings. He was treated chiefly with the free exhibition of opiates, beef-tea, and small quantities of wine occasionally; the wound being dressed daily with resinous ointment.

On the 7th, he is reported to have taken "ten ounces of wine since yesterday morning; about midnight, having had no sleep, fifty drops of Tr. Opii were given, and he slept soundly for several hours; one natural stool; pulse 128, of moderate strength; tongue clean; feels much better.—Habeat Vin. rub. ℥xij., et Haust. Anodyn. p. r. n. An egg for dinner daily."

From this period, the cure went on progressively, the lad's looks improved daily, while the wound assumed a more healthy appearance; the discharge gradually diminished; and he may now be considered as out of danger.

In my comments upon this case, I considered it as illustrative of three important points: the spontaneous cessation of the hemorrhage, in cases where limbs have been torn from the trunk; the question of primary and secondary amputation; and, lastly, the mode of performing this operation at the shoulder-joint.

In illustration of the first point, I showed you the portion of the humeral artery which was removed along with the remains of the arm, the internal coat of which was in some points slightly lacerated, while the external coat was overstretched, and a considerable portion of it filled with coagulum. I referred, for further illustration of the state of arteries after accidents of this kind, to a case detailed in the nineteenth volume of the Edinburgh Medical Journal, by Mr. Lizars; and to a very valuable paper, containing indeed all the information which we possess on this interesting subject, by Professor Turner, in the Medico-Chirurgical Transactions of this place. By the kindness of this last-mentioned gentleman, I was also enabled to show you some drawings, exhibiting the state of arteries in accidents of this nature; where, in some cases, we find their internal coats completely torn through, and corrugated, or coiled up, as it were, within the vessel, so as to prevent the effusion of blood.

On the subject of primary and secondary amputation, we now possess a series of valuable observations, from the time at which it was made the subject of a prize-question by the French Academy in 1756, down to the present day; when the advantages of primary amputation are, I believe, admitted by every practical surgeon, who is now enabled to add to his own experience that of the French army surgeons, as detailed in Baron Larrey's writings; that of the English army surgeons during the peninsular war, as detailed by Mr. Guthrie and Dr. Hennen; and that of the naval surgeons, as detailed in Mr. C. Hutchison's work. One of the most striking illustrations of the successful issue of primary amputations, is contained in an extract from a report made by Dr. Burke, inspector of hospitals to his

Majesty's forces in Bengal, which has been made public by my friend, Mr. ANNESLEY, in his splendid work on the Diseases of India. Dr. Burke states that, "of eighty cases of amputation," performed at Bhurtpore in Upper India, "the whole recovered in fourteen days."

We must not, however, expect to meet with the same proportional success in the amputations performed in civil hospitals: at least, I am entitled to say, that the contrast between the success of primary and secondary amputations, during the time that I have served in this house, has by no means resembled that which I was accustomed to see and hear of in the army. For these different results in military and in civil hospitals, many reasons might, I think, be assigned; some of them more satisfactory than those mentioned by SANSON, who, in a recent paper upon this subject, has noticed the fact, but has, I think, failed to give a very luminous or satisfactory explanation of it.

In speaking of the operation at the shoulder-joint, I remarked that, although this operation has undergone many modifications, to some of which the names of Morand, La Faye, Le Dran, Larrey, Lisfranc, Broomfield, Alanson, and others, have been attached, yet these may all be resolved into two modes of proceeding, either by forming a superior and inferior, or an anterior and posterior flap. I have myself operated in both ways, but not sufficiently often to enable me to institute any fair comparison as to the best mode of operating: I may, however, be permitted to remark that, in a very large proportion of the cases requiring amputation at the shoulder-joint, the soft parts are so lacerated as to leave us no choice, but to compel us, as in the present case, to form the flaps as circumstances best will admit. We are now well aware that the apprehension of an uncontrollable hemorrhage, which alarmed our predecessors, and made them slow to adopt this operation, is altogether unfounded: the bleeding may always be controlled by firm pressure above the clavicle by the hands of a steady assistant; and, in fact, this compression, as my distinguished predecessor, Dr. THOMSON, observes, has been found "easier in practice than it appeared to be in speculation." It is necessary, however, to remark, that, at the moment of cutting through the axillary vessels and nerves, the patient is apt to give an involuntary start, and may throw the fingers of the assistant off the artery; an accident which once happened to a gentleman assisting me in this operation, and by which I nearly lost my patient. I was for a moment completely blinded by the discharge of blood into my eyes from the open axillary artery.

Another case in which primary amputation was thought advisable during the present course, was that of Charles Cuthbertson, æt. seventy-one. This old man was admitted on the 18th of December, with a comminuted fracture of the radius and ulna at their carpal extremities, and an extensive lacerated wound of the left wrist. He had also received a severe contusion in the lower part of the back, with a fracture of some of the ribs. Although (as I stated to you in the theatre at the time of the operation,) I entertained little or no expectation of this man's recovery, I determined, after a few moment's consultation with my colleagues, to remove the arm a little below the elbow. It was obvious, however, at the first dressing, that no prospect of union existed: the lips of the wound, instead of presenting that wholesome turgidity and tension which presents itself in a healthy stump, were flaccid, somewhat livid, and apparently tending to gangrene; the smaller vessels (if I may use the expression,) had not taken up, but had poured out a quantity of grumous blood, which flowed through the dressings; at the same time the patient began to complain of oppressed breathing and symptoms of inflammatory action within the thorax; so that he had to contend with all those difficulties which you may suppose to exist in the case of an old man labouring under acute inflammation within the trunk, and gangrene in one of the extremities. Amidst a choice of evils, the abstraction of a limited quantity of blood was tried, but without any good effect, and the patient died during my temporary absence in London, about eight days after the receipt of the injury. No report of the dissection appears to have been entered in the register; but I am told that the whole of the ribs on the right side, with the exception of the two inferior ones, were found fractured, and some recent deposition of lymph on the pleuræ.

This case I am induced to mention, not from any very instructive lesson to be gathered from its progress, much less for any thing very unexpected in its event, but as a caution against an accident which occurred during the operation. While passing the knife through the forearm, from the radial to the ulnar side, for the purpose of forming a posterior flap from the extensor and supinator muscles, its point slipped between the bones, which rendered it necessary to withdraw it, and to pass it more carefully behind the ulna: this was, perhaps, owing partly to my own inadvertence to the exact position of the bones, but partly, I believe also, to the connexion between the lower extremities of the radius and ulna having been broken up, so that, in pressing the knife close to the back of the radius, with the view of obtaining a sufficiency of muscular substance to form a good flap, the parallelism of the two bones was in some measure destroyed, the radius was pressed forwards, and the interosseous space thus presented to the point of the knife.

[To be continued.] 95.

